# Higher Hospitalization Rate for Lower Airway Infection in Transfusion-Naïve Thalassemia Children

**DOI:** 10.3389/fped.2020.574014

**Published:** 2020-11-24

**Authors:** Ti-An Tsai, Chang-Ku Tsai, Yao-Hsu Yang, Zon-Min Lee, Jiunn-Ming Sheen, Yi-Chen Lee, Chih-Min Tsai, Chih-Cheng Chen, Chih-Hao Chang, Chen-Kuang Niu, Hong-Ren Yu

**Affiliations:** ^1^Department of Pediatrics, Chang Gung Memorial Hospital-Kaohsiung Medical Centre, Kaohsiung City, Taiwan; ^2^Department of Chinese Medicine, Chiayi Chang Gung, Chiayi City, Taiwan; ^3^Department of Pharmacy, Kaohsiung Chang Gung Memorial Hospital, Kaohsiung City, Taiwan; ^4^Department of Respiratory Therapy, Chang Gung Memorial Hospital-Kaohsiung Medical Centre, Kaohsiung City, Taiwan; ^5^Graduate Institute of Clinical Medical Sciences, College of Medicine, Chang Gung University, Taoyuan City, Taiwan

**Keywords:** thalassemia, transfusion-naïve, pneumonia, bronchitis, children

## Abstract

Few studies have addressed the risk of infection in transfusion-naïve thalassemia patients. We aimed to investigate whether transfusion-naïve thalassemia population has higher hospitalization rates for lower airway infection-related diseases than non-thalassemia population in children. A nationwide population-based retrospective cohort study was conducted using detailed medical records of the Taiwan National Health Insurance Research Database. Transfusion-naïve thalassemia patients were compared with a matched cohort at a ratio of 1:4. Data of the selected patients were adjusted for age, sex, and related comorbidities. We recorded the frequency of admissions or outpatient clinic visits for patients with a diagnosis of pneumonia or acute bronchitis/bronchiolitis. Based on our results, the hospitalization rates and incidence rate ratios of bronchitis/bronchiolitis and pneumonia for transfusion-naïve thalassemia children were all higher than those for non-thalassemia controls. Therefore, we conclude that transfusion-naïve thalassemia children are more likely to experience lower airway infections and have a higher probability of hospitalization for these conditions.

## Introduction

Thalassemia is a common autosomal-recessive hereditary hemoglobinopathy, mainly characterized by a point mutation on globin gene expression (1). In adults, hemoglobin comprises four protein chains (two α and two β globin chains) that are assembled into a heterotetramer. In α-thalassemia and β-thalassemia, the production of the α and β globin chains is affected, respectively. Thalassemia is commonest among people of Italian, Greek, Middle Eastern, South Asian, and African descent (2). In China, the prevalence of α-thalassemia, β-thalassemia, and α + β-thalassemia were estimated at 7.88, 2.21, and 0.48%, respectively (3). In a previous study, the prevalence of α-thalassemia traits in Taiwan was 3.4% (4).

Clinical manifestations of thalassemia may range from no symptoms to severe lethal complications depending on how many of the four genes for α globin or two genes for β globin are involved. People with thalassemia may have iron overload (owing to the disease itself or frequent blood transfusions), infection, splenomegaly, slowed growth rates, or heart disease. Infection is a major cause of morbidity and mortality in patients with thalassemia major and is assumed to result from splenectomy, blood transfusion, and immunological changes with iron overload (2, 5). Patients with splenectomy are more susceptible to gram-negative microorganisms and pneumococcus than other organisms (6, 7), and patients who receive regular blood transfusions are at a risk of developing transfusion-transmitted infections such as *Toxoplasma gondii*, hepatitis C virus, and hepatitis B virus (8–10).

Unlike thalassemia major, patients with non-transfusion-dependent thalassemia (NTDT) (including those with various phenotypes) do not require lifelong regular transfusion therapy for survival. NTDT patients can be at risk of ineffective erythropoiesis, peripheral hemolysis, and iron overload that contribute to a number of clinical morbidities (11, 12), and these patients with iron overload may have higher risk for severe bacterial infection (13).

Intracellular iron overload has been proven to lead to DNA damage of lymphocytes and immune dysfunction in thalassemia major patients who receive blood transfusions (14). However, whether the immune function in NTDT patients is impaired is still unclear. Some reports suggested the presence of decreased CD4+/CD8+ ratios and increased Treg cells, which might suppress immune activation status, in β-thalassemia major patients, but not in those with β-thalassemia traits, compared to controls (15, 16). Another report stated that reduced CXCR2 expression and neutrophil migration were observed in NTDT patients (17). Therefore, this study aimed to examine whether NTDT children without history of blood transfusion have a higher risk of infection, especially lower respiratory tract infections.

## Methods

### Data Source

Nearly the entire Taiwanese population, i.e., over 23 million people, have been enrolled in National Health Insurance since 1995. Details of the medical records of each person are stored in the National Health Insurance Research Database (NHIRD), which was created by the National Health Research Institutes (NHRI). In this retrospective cohort study, we used the Longitudinal Health Insurance Database 2010 (LHID 2010), released by NHRI, as our data source. The LHID 2010 includes one million people randomly selected from the 2010 Registry for Beneficiaries. The database contains detailed medical records of each insurant from 1997 to 2013. Among these one million people, we selected people whose birth year was from 1998 to 2007, so all people we analyzed were children and adolescents. International Classification of Diseases, ninth revision (ICD-9-CM) coding was used for disease identification. The study protocol was approved by the institutional review board of the study hospital.

### Sampled Participants

We defined patients as having thalassemia if they were diagnosed with thalassemia (ICD-9-CM: 282.4) once during hospitalization or at least twice when examined at the outpatient department in 1 year. We excluded patients with a history of catastrophic illness certificates, blood transfusion (ICD-9 procedure code 94005C or 990), partial or total splenectomy (ICD-9 procedure code 70001B, 70003B, and 70006B), immunodeficiency (ICD-9-CM: 279) and other hematological disorders, including iron deficiency, and other deficiency anemias (ICD-9-CM: 280~281), sickle-cell anemia and other hemoglobinopathies, hereditary/acquired hemolytic anemia, aplastic anemia, and chronic disease-related anemia (ICD-9-CM: 282.6~285.8), myelodysplastic syndrome (ICD-9-CM: 238.7), hyperferritinemia and primary or secondary hemochromatosis (ICD-9-CM: 275.0), and hematological malignancies (ICD-9-CM: 200~208) diagnosed once during hospitalization or more than once in the outpatient department in 1 year. We also excluded patients for whom information on age or sex was missing. The catastrophic illness certificates in Taiwan include cancer, hematologic abnormality, renal failure with hemodialysis required, generalized autoimmune diseases with life-long treatment required, chronic mental disorders, congenital metabolic disorders, major organs and genes abnormality (e.g., congenital anomalies of heart), massive burns, major organs transplantation, complicated nervous, or musculoskeletal disorders (e.g., cerebral palsy), injury severity scores more than 16, chronic respiratory failure with ventilator required, uncorrected malnutrition status, Myasthenia gravis, spinal cord injuries, occupational diseases, acute stage of cerebrovascular diseases, multiple sclerosis, leprosy, liver cirrhosis with complication, complications related to prematurity, toxic effect of arsenic and its compounds, Creutzfeldt-Jakob disease, and other rare diseases (e.g., cystic fibrosis).

One thalassemia patient was matched with four control patients without thalassemia (1:4 matching) according to birth year, sex with frequency match. The comorbidities included were asthma (ICD-9-CM: 493), diabetes mellitus (DM) (ICD-9-CM: 450), chronic lung disease (ICD-9-CM: 770.7), heart failure (ICD-9-CM: 428), epilepsy (ICD-9-CM: 345), and chronic kidney disease (CKD) (ICD-9-CM: 580~589), diagnosed once at hospitalization or at least thrice in the outpatient department in 1 year. These medical conditions were proved to be risk factors of pneumonia (18, 19). The propensity score was calculated using the Statistical Analysis System 9.4 program (SAS Institute, Cary, North Carolina, USA).

### Outcome and Comorbidities

The patients in this cohort study were followed up from their birthday to death or 2013/12/31. The outcome was defined as having records of lower airway infections such as acute bronchitis/bronchiolitis (ICD-9-CM: 466) and pneumonia (ICD-9-CM: 480~486), diagnosed at hospitalization or at least thrice in the outpatient department in 1 year. Each incident of hospitalization due to lower respiratory tract infection was included for analysis. The effects of baseline comorbidities, which may be related to lower airway infection, were eliminated via the propensity score model.

### Statistical Analysis

Differences between the demographic characteristics and comorbidities and the events of hospitalization due to lower airway infections in patients with thalassemia and matched non-thalassemia controls were analyzed using the chi-square test. The incidence rate ratios (IRRs) and 95% confidence intervals (CIs) were estimated using Poisson regression. A *P* < 0.05 in 2-tailed tests was considered statistically significant.

## Results

**Figure 1:**
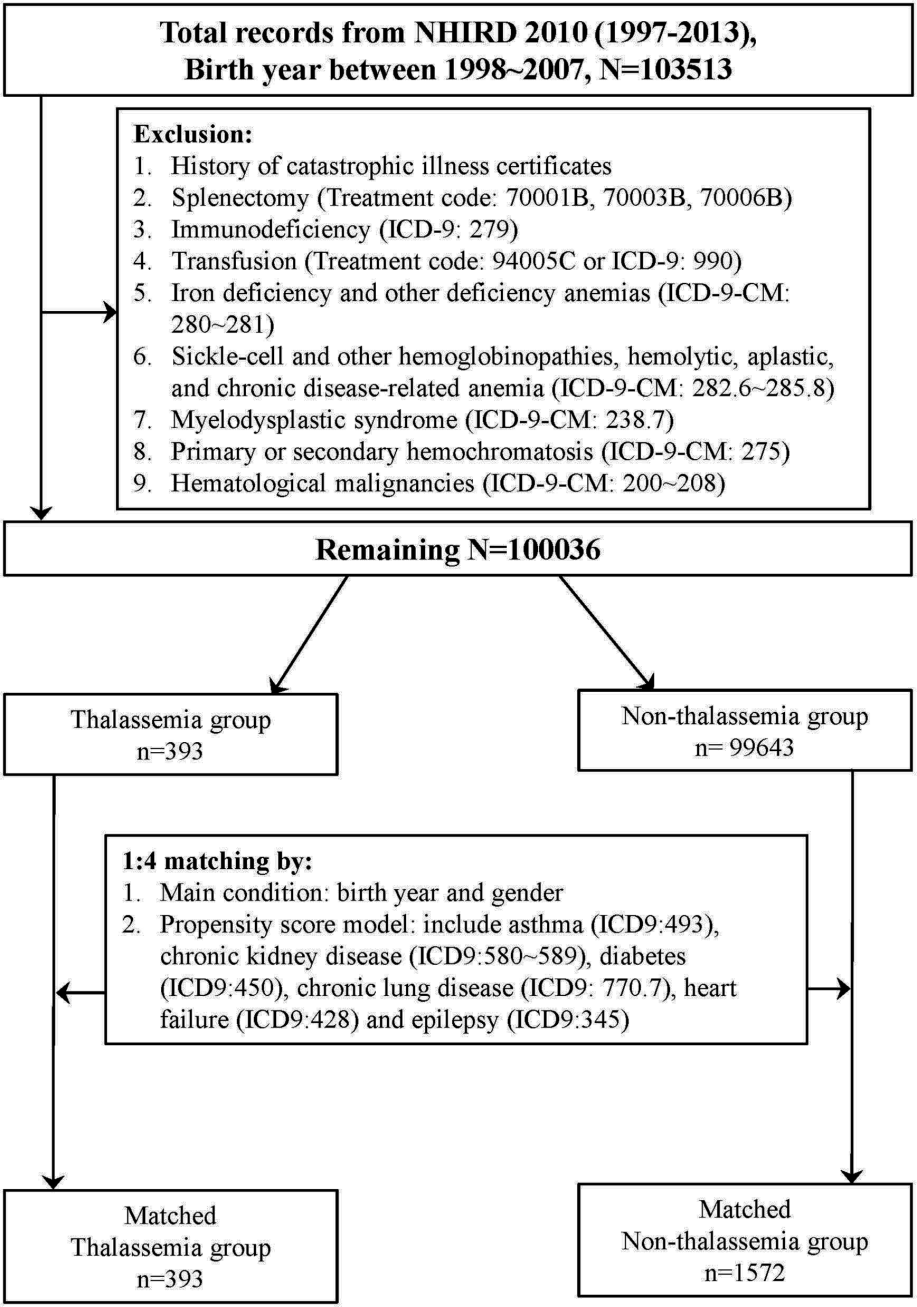
Flow chart of matched cohorts' selection. One million people were randomly selected from the Longitudinal Health Insurance Database 2010 (LHID 2010). After the screening process, 393 persons in the thalassemia group and 1,572 persons in the non-thalassemia group were analyzed.

One million people were randomly selected from LHID 2010, and those born between 1998 and 2007 were selected for further analysis. Subjects who matched the inclusion and exclusion criteria were separated into a group with thalassemia and a group with non-thalassemia. Details of these processes are shown in Figure 1. Finally, 393 patients were identified in the thalassemia group and 1,572 controls in the non-thalassemia group (the control group), with 1:4 matching according to birth year, sex, and propensity score.

Demographic characteristics and comorbidities in these matched cohorts are shown in Table 1. Similar distributions occurred with sex, age, place of residence, income, and comorbidities, including asthma, DM, CKD, chronic lung disease, heart failure, and epilepsy between the thalassemia and non-thalassemia groups because both cohorts were matched for factors. For lower respiratory diseases, the patients in the thalassemia group had a higher prevalence of acute bronchiolitis/bronchitis (92.88% vs. 87.60%, *P* = 0.003) and pneumonia (60.65 vs. 36.96%, *P* < 0.001) than those in the non-thalassemia group.

**Table 1:** Demographic characteristics and comorbidities in matched cohorts.

			**THA**, ***n*** **=** **393 (20%)**		**Non-THA**, ***n*** **=** **1572 (80)**		
***N* = 1,965**		***N* (%)**	***n***	**%**	***n***	**%**	***P***
Gender	Male	1205	241	61.32	964	61.32	1
	Female	760	152	38.68	608	38.68	
Birth year	1998	175	35	8.91	140	8.91	1
	1999	230	46	11.7	184	11.7	
	2000	255	51	12.98	204	12.98	
	2001	215	43	10.94	172	10.94	
	2002	200	40	10.18	160	10.18	
	2003	150	30	7.63	120	7.63	
	2004	160	32	8.14	128	8.14	
	2005	205	41	10.43	164	10.43	
	2006	175	35	8.91	140	8.91	
	2007	200	40	10.18	160	10.18	
Comorbidity	Yes	760	152	38.68	608	38.68	1
	No	1205	241	61.32	964	61.32	
Asthma	Yes	710	142	36.13	568	36.13	1
	No	1255	251	63.87	1004	63.87	
CKD	Yes	25	5	1.27	20	1.27	1
	No	1940	388	98.73	1552	98.73	
Diabetes	Yes	0					1
	No	1965	393	100	1572	100	
CLD	Yes	0					1
	No	1965	393	100	1572	100	
Heart failure	Yes	0					1
	No	1965	393	100	1572	100	
Epilepsy	Yes	40	8	2.04	32	2.04	1
	No	1925	385	97.96	1540	97.96	
Bronchiolitis	Yes	1742	365	92.88	1377	87.6	0.003
	No	223	28	7.12	195	12.4	
Pneumonia	Yes	817	236	60.05	581	36.96	<0.001
	No	1148	157	39.95	991	63.04	

*Chi-square test*.

*THA, thalassemia; Non-THA, non-thalassemia; CLD, chronic lung disease; CKD, chronic kidney disease*.

**Figure 2:**
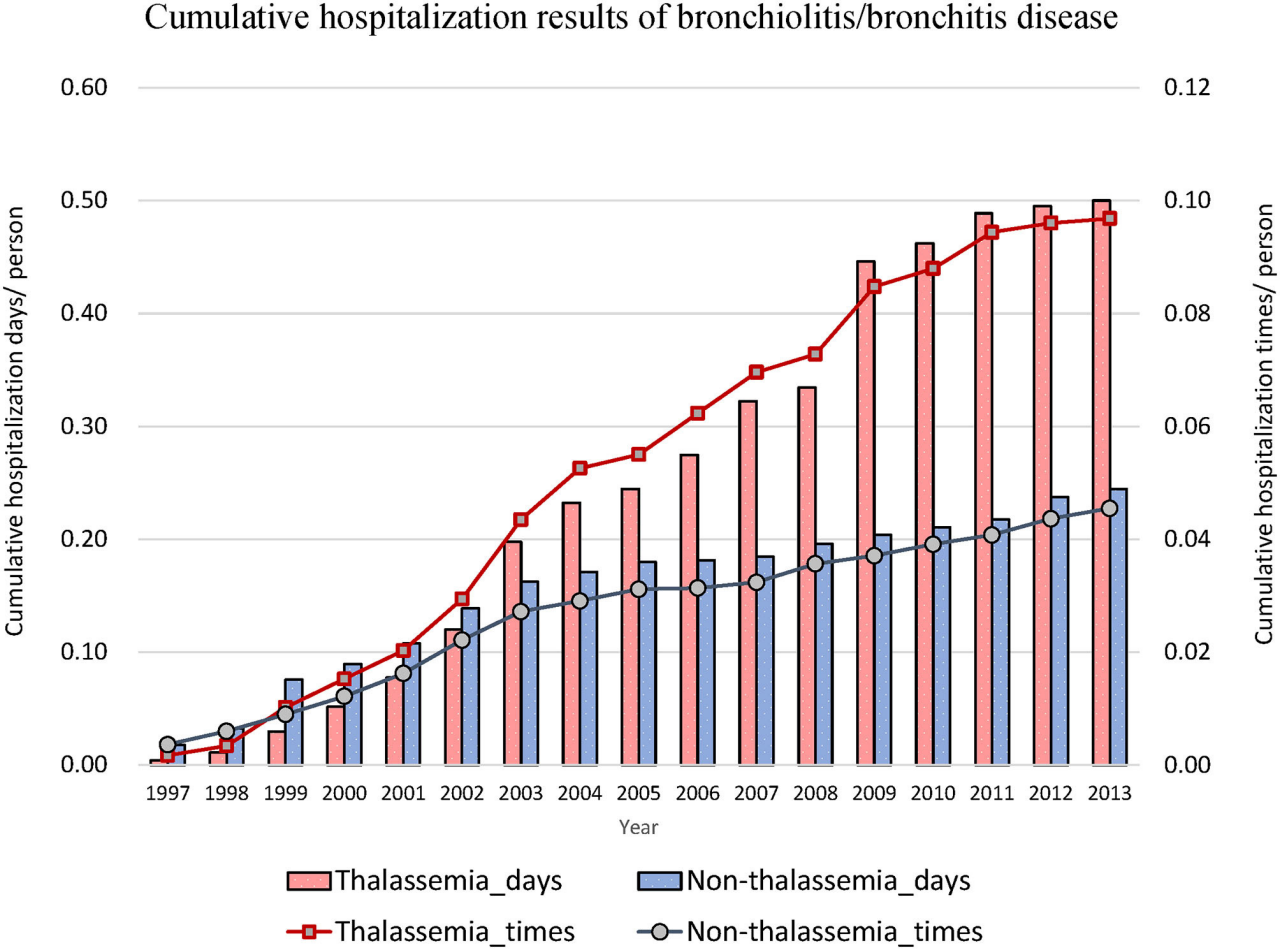
Cumulative number of admissions due to acute bronchitis/bronchiolitis for patients with (dashed line) or without (solid line) thalassemia, and cumulative hospitalization days for patients with (white bar) or without (black bar) thalassemia. The x-axis is the cumulated years from 1997 to 2013, 17 years in total. The cumulative rate for the thalassemia group was higher than that for the non-thalassemia group.

**Figure 3:**
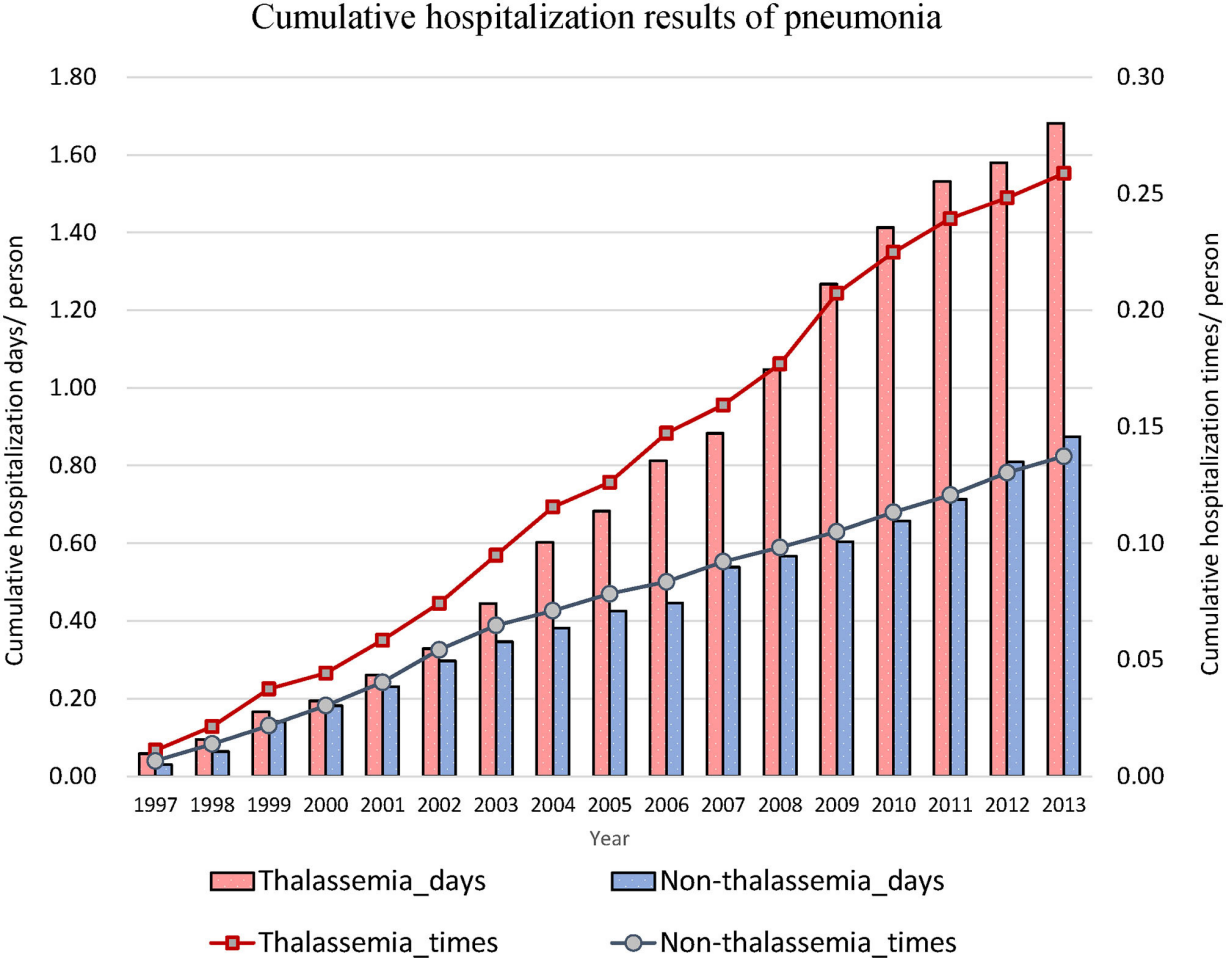
Cumulative number of admissions due to pneumonia for patients with (dashed line) or without (solid line) thalassemia, and cumulative hospitalization days for patients with (white bar) or without (black bar) thalassemia. The x-axis is the cumulated years from 1997 to 2013, 17 years in total. The cumulative rate for the thalassemia group was higher than that for the non-thalassemia group.

Thereafter, we focused on events related to hospitalization due to lower respiratory diseases (Table 2). The hospitalization rates for acute bronchitis/bronchiolitis (31.55 vs. 13.42%, *P* < 0.001) and pneumonia (49.11 vs. 23.22%, *P* < 0.001) were also higher in the thalassemia group than in the non-thalassemia group. The average number of admissions for inpatients and the average number of hospitalization days each time were also compared, and there was almost no difference between these two groups. Figures 2, 3 show the cumulative number of admissions or hospitalization days per person due to acute bronchitis/bronchiolitis and pneumonia across the 17 years, respectively. For both acute bronchitis/bronchiolitis and pneumonia, the cumulative rates for the thalassemia group were obviously higher than for the non-thalassemia group.

**Table 2:** Number of inpatients, number of admissions and hospitalization days for lower airway disease (acute bronchitis/bronchiolitis and pneumonia) from matched cohorts.

		**Overall**	**THA**, ***n*** **=** **393**		**Non-THA**, ***n*** **=** **1,572**		***P***
BRO	Inpatients, *n* (%)	335	124	(31.55)	211	(13.42)	<0.001^a^
	Total admissions	501	208		293		
	Total hospitalization days	2,299	939		1,360		
	Total admissions/Total inpatients	1.50 ± 1.11^*^	1.68 ± 1.42^*^		1.39 ± 0.87^*^		0.0417^b^
	Total days/Total admissions	4.59 ± 2.47^*^	4.51 ± 2.70^*^		4.64 ± 2.30^*^		0.5811^b^
PN	Inpatients, *n* (%)	558	193	(49.11)	365	(23.22)	<0.001^a^
	Total admissions	926	354		572		
	Total hospitalization days	4,301	1,753		2,548		
	Total admissions/Total inpatients	1.66 ± 1.30^*^	1.83 ± 1.51^*^		1.57 ± 1.16^*^		0.0329^b^
	Total days/Total admissions	4.64 ± 2.61^*^	4.95 ± 2.70^*^		4.45 ± 2.54^*^		0.0048^b^

* *Plus-minus values are mean ± SD*.

a *T-test*.

b *Chi-square test*.

*THA, thalassemia; Non-THA, non-thalassemia; BRO, acute bronchitis/bronchiolitis; PN, pneumonia*.

The IRR of admissions due to acute bronchitis/bronchiolitis and pneumonia was also analyzed (Tables 3, 4, respectively). Age of the study patients was classified into three groups: preschooler (<6 years); grade schooler (≥6 to <12 years); and teenager (≥12 to <16 years). In Tables 3, 4, “Age (year old)” implied the age of the patient at the date of admission, and “Times” implied the number of admissions. Person-years (PY) was calculated as:

$$\begin{array}{l} \text{PY}=\left[\text{deathdateor}2013/12/31\right]-\left[\text{birthday}\right]. \end{array}$$

For acute bronchitis/bronchiolitis, those with transfusion-naïve thalassemia had a higher incidence rate of admission than the non-thalassemia controls, irrespective of sex, age, or whether there was comorbidity or not. The only exception was patients with epilepsy or chronic kidney diseases, but these were not statistically significant because only few data were included for analysis. Comparing the age at admission, grade schoolers (≥6 to <12 years) had higher IRR (IRR 4.48, 95% CI 2.64–7.61) than preschoolers. The IRR of teenagers could not be calculated because no non-thalassemia teenagers were admitted for acute bronchiolitis/bronchitis during our study period. The admissions due to pneumonia showed the same trend. Those with transfusion-naïve thalassemia had higher incidence rates of admissions than non-thalassemia controls, irrespective of sex, age, or presence of comorbidity, except for the patients with chronic lung diseases or epilepsy. The highest IRR according to age of admission was observed in the teenager group (IRR 23.98, 95% CI 2.89–199.22). Most inpatients who were admitted for acute bronchitis/bronchiolitis or pneumonia were aged below 6-year-old.

**Table 3:** Incidence rate ratio of admissions for acute bronchitis/bronchiolitis.

	**THA (*****n*** **=** **393)**				**Non-THA (*****n*** **=** **1,572)**					
	***N***	**Times**	**PY**	**Rate (per 1,000 PY)^a^**	***N***	**Times**	**PY**	**Rate (per 1000 PY)^a^**	**IRR (95% CI)^†^**	***p***
Overall	124	208	4393.12	47.35 (41.33–54.24)	211	293	17602.2	16.65 (14.84–18.67)	2.84 (2.38–3.4)	^***^
Female	42	74	1662.83	44.50 (35.43–55.89)	78	100	6670.8	14.99 (12.32–18.24)	2.97 (2.2–4.01)	^***^
Male	82	134	2730.28	49.08 (41.43–58.13)	133	193	10931.4	17.66 (15.33–20.33)	2.78 (2.23–3.47)	^***^
Age (y/o) <6	112	176	2358	74.64 (64.39–86.52)	197	267	9432	28.31 (25.11–31.92)	2.64 (2.18–3.19)	^***^
6≦Age <12	21	29	1692.02	17.14 (11.91–24.66)	20	26	6798.7	3.82 (2.60–5.62)	4.48 (2.64–7.61)	^***^
12≦Age <16	2	3	343.1	8.74 (2.82–27.11)	0	0	1371.49	0.00		
No comorbidity	56	84	2654.97	31.64 (25.55–39.18)	97	119	10630.7	11.19 (9.35–13.4)	2.83 (2.14–3.74)	^***^
Comorbidity^b^	68	124	1738.15	71.34 (59.83–85.07)	114	174	6971.46	24.96 (21.51–28.9)	2.86 (2.27–3.6)	^***^
w/o Asthma	57	85	2774.88	30.63 (24.77–37.89)	103	127	11115	11.43 (9.60–13.6)	2.68 (2.04–3.53)	^***^
with Asthma	67	123	1618.24	76.01 (63.70–90.70)	108	166	6487.16	25.59 (21.98–29.7)	2.97 (2.35–3.75)	^***^
w/o CKD	124	208	4328.54	48.05 (41.95–55.05)	206	285	17339.9	16.44 (14.63–18.4)	2.92 (2.45–3.5)	^***^
with CKD	0	0	64.58	0.00	5	8	262.31	30.50 (15.25–60.9)	0	^***^
w/o Epilepsy	123	207	4293.28	48.21 (42.07–55.25)	207	287	17198.7	16.69 (14.86–18.7)	2.89 (2.42–3.45)	^***^
with Epilepsy	1	1	99.84	10.02 (1.41–71.11)	4	6	403.48	14.87 (6.68–33.1)	0.67 (0.08–5.59)	0.715

† *Poisson regression*,

*** *p < 0.001*.

a *Rate, incidence rate, per 1,000 person years*.

b *Individuals with any comorbidity of asthma, epilepsy, and chronic kidney disease*.

*THA, thalassemia; Non-THA, non-thalassemia; PY, person-year; IRR, incidence rate ratio; CI, confidence interval; w/o, without; CKD, chronic kidney disease*.

**Table 4:** Incidence rate ratio of admissions for pneumonia.

	**THA (*****n*** **=** **393)**				**Non-THA (*****n*** **=** **1,572)**					
	***N***	**Times**	**PY**	**Rate (per 1,000 PY)^a^**	**N**	**Times**	**PY**	**Rate (per 1,000 PY)^a^**	**IRR (95% CI)^†^**	***p***
Overall	193	354	4393.12	80.58 (72.61–89.43)	365	572	17602.2	32.50 (29.94–35.27)	2.48 (2.17–2.83)	^***^
Female	71	131	1662.83	78.78 (66.38–93.50)	135	198	6670.8	29.68 (25.82–34.12)	2.65 (2.13–3.31)	^***^
Male	122	223	2730.28	81.68 (71.63–93.13)	230	374	10931.4	34.21 (30.92–37.86)	2.39 (2.02–2.82)	^***^
Age (y/o) <6	178	305	2358	129.35 (115.62–144.71)	332	490	6432	76.18 (47.55–56.76)	2.49 (2.16–2.87)	^***^
6≦Age <12	31	43	1692.02	25.41 (18.85–34.27)	60	81	6798.7	11.91 (9.58–14.81)	2.13 (1.47–3.09)	^***^
12≦Age <16	3	6	343.1	17.49 (7.86–38.93)	1	1	1371.49	0.73 (0.10–5.18)	24 (2.89–199.22)	0.003
No comorbidity	106	161	2654.97	60.64 (51.96–70.77)	154	200	10630.7	18.81 (16.38–21.61)	3.22 (2.62–3.97)	^***^
Comorbidity^b^	87	193	1738.15	111.04 (96.43–127.86)	211	372	6971.46	53.36 (48.20–59.07)	2.08 (1.75–2.48)	^***^
w/o Asthma	109	164	2774.88	59.10 (50.71–68.88)	162	214	11115	19.25 (16.84–22.01)	3.07 (2.5–3.76)	^***^
with Asthma	84	190	1618.24	117.41 (101.85–135.35)	203	258	6487.16	39.77 (49.76–61.21)	2.13 (1.78–2.54)	^***^
w/o CKD	192	353	4328.54	81.55 (73.47–90.52)	358	553	17339.9	31.89 (29.34–34.66)	2.56 (2.24–2.92)	^***^
with CKD	1	1	64.58	15.48 (2.18–109.93)	7	19	262.31	72.43 (46.20–113.56)	0.21 (0.03–1.6)	^***^
w/o Epilepsy	190	351	4293.28	81.76 (73.63–90.77)	356	556	17198.7	32.33 (29.75–35.13)	2.53 (2.21–2.89)	^***^
with Epilepsy	3	3	99.84	30.05 (9.69–93.17)	9	16	403.48	39.66 (24.29–64.73)	0.76 (0.22–2.6)	0.659

† *Poisson regression*,

*** *p < 0.001*.

*THA, thalassemia; Non-THA, non-thalassemia; PY, person-year; IRR, incidence rate ratio; CI, confidence interval; w/o, without; CKD, chronic kidney disease*.

a *Rate, incidence rate, per 1,000 person years*.

b *Individuals with any comorbidity of asthma, epilepsy, and chronic kidney disease*.

## Discussion

Thalassemia is a common hematologic disorder, and its complications include bone dysmorphism, hemochromatosis, splenomegaly, infection, endocrine disorders, and heart failure. Some of these complications result from long-term, repeated blood transfusions (20). Infection is a major issue for patients with the severe form of thalassemia, and the proposed predisposing factors are anemia, iron overload, splenectomy, transfusion-associated viral infections, and a range of immune abnormalities (2). For thalassemia patients without a history of blood transfusion, although there may be no symptoms of anemia or it may be mild, such as thalassemia minor patients, they also have a higher risk of coronary artery disease, erectile dysfunction, and fractures (21–23). Their hematopoiesis may be less effective, and the abnormal erythrocyte cell membrane structure leads to peripheral hemolysis and subsequent iron overload, which are risk factors for susceptibility to infection (11, 12). However, no strong evidence to this effect has been provided before our study.

The risk of infection for thalassemia major patients has been demonstrated in previous studies; our study, a nationwide, population-based cohort study, revealed that transfusion-naïve thalassemia children are also susceptible to lower respiratory tract infection. This present study showed a higher prevalence of acute bronchitis/bronchiolitis and pneumonia, common diseases related to lower respiratory infection, in the transfusion-naïve thalassemia population compared to the non-thalassemia controls. Previous studies had shown that non-transfusion-dependent thalassemia (NTDT) patients may have altered immune function and increased susceptibility to severe bacterial infections, especially for patients with iron overload or post splenectomy (13, 17). The NTDT patients do not need lifelong regular blood transfusion but occasional blood transfusion may be required for some stressful conditions. Patients who had ever received blood transfusion, splenectomy, or had been diagnosed as having other hematologic disorders were excluded based on the ICD-9-CM codes in this study. Thus, our study group is in a milder status than NTDT. We assume that the thalassemia patients that included are silent carriers and have α- or β- thalassemia minor or α- or β-thalassemia intermedia without a history of blood transfusion.

The effects of confounding factors, i.e., age, sex, and comorbidities of asthma, heart failure, DM, chronic lung disease, epilepsy, and CKD, were adjusted for when matching the thalassemia and control groups. Focusing on those who were hospitalized with diseases related to lower respiratory infection, the thalassemia group had a higher percentage of admissions for both acute bronchitis/bronchiolitis and pneumonia compared to the control group. However, there was almost no difference between the two groups with respect to average number of admissions and average days of hospitalization of inpatients. It seemed that although transfusion-naïve thalassemia patients had a higher possibility of hospitalization than the non-thalassemia controls, irrespective of sex, age, or presence of comorbidity, the severity of the diseases was similar. In this study, most inpatients were aged below 6 years, which may be because the immune system of preschoolers is relatively immature (24, 25), and to some extent, the criterion of admission maybe loose. Among different age groups, the highest IRR fell into the teenager (≥12 to <16 years).

Similar to previous similar studies, this study has several limitations (21–23). First, there are several genotypes of thalassemia, in which the structure of hemoglobin is different. Thus, thalassemia patients have varying hemoglobin and ferritin levels. These data may affect the risk of infection and they could not be obtained from the NHIRD. However, the proportion of thalassemia intermedia is just about 1.5 percent among the NHIRD (26). We had also excluded cases that received blood transfusion and splenectomy, which account for near 50% and 30 of thalassemia intermedia (27). Thus, the effect of thalassemia intermedia in NHIRD is too small and can be neglected. Second, although we used ICD-9 codes to screen out thalassemia patients, some asymptomatic patients were missed because the physicians did not include thalassemia in the diagnostic lists for this population. There might have been some thalassemia patients in the control group. Thus, the hospitalization rate for lower airway infection in the control group may be overestimated. In other words, in real condition, the NHIRD may have higher hospitalization rates for lower airway infection than control. The incidence of pneumococcus disease decreased dramatically after the introduction of pneumococcal vaccine (28). In Taiwan, pneumococcal vaccine has been provided for free since 2015 to all young children (29), which was beyond our study period (1997–2013). The introduction of pneumococcal vaccine should have no influence on our data.

Nowadays, the number of cases of severe thalassemia in Taiwan is decreasing because of the policy of prenatal screening. Most thalassemia patients are silent carriers or have thalassemia minor. Previously the prevention of infections was usually focused on thalassemia patients with history of blood transfusion. The main contribution of our study is the elucidation of the risk of lower airway infection in transfusion-naïve thalassemia patients. It is therefore important to identify the nearly asymptomatic thalassemia populations and educate them regarding the importance of preventing themselves from acquiring airborne or droplet-transmitted infections.

The phenomenon that transfusion-naïve thalassemia patients are susceptible to lower respiratory tract infection was observed in our study, and this observation proved that besides of blood transfusion, there are other mechanisms contributes to higher infection risk, like ineffective erythropoiesis and increased intestinal iron absorption (30), although the exact pathologic mechanism remains unclear. Further studies should be designed to obtain more details on the pathophysiology concerned. In addition, it would be valuable to study whether these transfusion-naïve thalassemia patients have a higher risk of other types of infection, such as urinary tract infection and acute gastroenteritis.

## Data Availability Statement

The datasets presented in this study can be found in online repositories. The names of the repository/repositories and accession number(s) can be found in the article/supplementary material.

## Ethics Statement

The studies involving human participants were reviewed and approved by Chang Gung Memorial Hospital-Kaohsiung Medical Center. Written informed consent for participation was not provided by the participants' legal guardians/next of kin because: big data (Taiwan National Health Insurance Research Database).

## Author Contributions

H-RY, Z-ML, T-AT, and C-KT contributed to design of the work. H-RY, Y-HY, Y-CL, and T-AT contributed to data acquisition. H-RY, C-KT, Y-CL, C-CC, and C-MT performed data analysis and interpretation. H-RY, C-MT, C-HC, and C-KN drafted the manuscript. H-RY, Z-ML, T-AT, C-KT, and C-KN finalized the article. All authors have read and approved the final manuscript and agreed to be accountable for all aspects of the work.

## Conflict of Interest

The authors declare that the research was conducted in the absence of any commercial or financial relationships that could be construed as a potential conflict of interest.
